# Letter from Vienna

**Published:** 1881-03

**Authors:** W. T. Belfield

**Affiliations:** Vienna


### Foreign Correspondence.

### ARTICLE X.

### LETTER FROM VIENNA.

VIENNA, FEBRUARY 6, 1881.

EDITORS CHICAGO MEDICAL JOURNAL AND EXAMINER:

I send you a brief account of a case which is the sensation of the hour in both medical and lay circles here, namely, a resection of the stomach. The operation was first proposed and successfully executed upon dogs seventy years ago by Merrem, and several pupils of Billroth have in recent years made extended researches as to its practicability upon man. The first actual operation upon a human being was made by Péan, in Paris, in 1879, for pyloric cancer, the patient dying on fourth day. Billroth has long been anxiously awaiting an opportunity which finally presented itself, two weeks ago, in the person of a woman forty-three years old, who had enjoyed excellent health until last October, when she began to exhibit symptoms of gastric cancer, and to suffer a corresponding decline in health. Upon examination by Billroth, a tumor as large as an orange was found at pylorus. Deeming the case a favorable one for operation, Billroth made a transverse incision upon tumor, which was found to be carcinoma of pylorus and of lower third of stomach. Adhesions with omentum and colon were carefully broken up—the large and small omenta cautiously separated. All vessels were ligated before division, so that the bleeding was very slight. The tumor was brought out and laid upon abdominal wall, and an incision made through stomach—at first only backwards—one centimeter beyond the infiltrated part. A similar incision was then made through the abdomen. Six stitches were applied to keep edges of wound together. The incision was then extended, completely severing the stomach. The edges were then united so as to leave an opening corresponding in size to the duodenum. The tumor was then completely separated from duodenum by an incision parallel with that in stomach. The edges of the two organs were then carefully adapted by means of fifty stitches with carbolized silk, and the entire wound cleansed with two per cent. carbolic acid solution. The operation, including narcosis, lasted one and a half hours. This was January 29. The patient has been entirely comfortable—but very slight fever, no pain. During the first days nutrition was maintained by clysters of pepsin and wine, but the woman can now digest milk comfortably. The prospects for recovery from the operation are excellent, as the edges must by this time have become thoroughly adherent. The piece removed was fourteen centimeters long; a goose quill was with difficulty passed through the pyloric orifice. The stomach is not materially changed in shape, though it is, of course, smaller.

W. T. BELFIELD.

---

THE PYGOPAGI.—The pygopagi is the curious appellation assigned to two young children now on exhibition at the Egyptian Hall, Piccadilly. They present features of singular interest, however, and are well worth inspection, as showing the limits within which it is possible to effect safe delivery of abnormally developed children. The pygopagi are two girls, firmly united at the pelvis, and being everywhere else distinct and separate. Into this conjunction certain of the external genitalia enter also, and how far this may be the case internally must, of course, be a matter of conjecture. Dr. Playfair has manifested considerable interest in the children, and proposes to exhibit them at the Obstetrical Society early in December. As a spectacle the two little objects present nothing to repulse, but are rather attractive in appearance, their movements and expressions being interesting and amusing. That they are perfectly independent in ideas and desires is evident, though they agree apparently in the best possible way. They are well developed for their age, a little over two years, and seem healthy and lively.

